# Atlantic Conjunctures in Anglo-American Neurology: Lewis H. Weed and Johns Hopkins Neurology, 1917-1942

**DOI:** 10.1353/bhm.0.0101

**Published:** 2008

**Authors:** Stephen T. Casper

**Keywords:** Britain-America, specialization-medicine, neurology, psychiatry, Rockefeller Foundation, global history, transnational

## Abstract

The emergence of neurology at Johns Hopkins presents a case study for reconsidering the international and institutional contexts of neurology generally. Using a variety of sources, Hopkins's interwar plans for neurology are presented and contextualized in the international environment of neurology, medical research, and philanthropy. During this period, neurology across the world, especially in Britain, was undergoing vast institutional changes. In order for Hopkins to remain at the forefront of excellence in both medicine and medical education, a program in neurology was deemed essential, and this would seem now to have been an unproblematic advance. Spearheading the project for the establishment of neurology at Hopkins was the dean of the medical school, Lewis H. Weed. Weed attempted from 1919 until 1942 to establish a department of neurology but had only limited success. The fact that finding support proved challenging for Weed and Johns Hopkins casts a provocative light on the broader historiography of neurology and illustrates the important role of the international context in defining neurology professionally.

Though historians have traditionally identified the emergence of neurology only with the production of neurological knowledge in the nineteenth century and the discoveries of individual physicians and scientists,^1^ some scholars are now arguing that clinical neurology emerged as a distinct specialist field of practice in the interwar period.^2^ The reasons for this revisionist view are varied. Despite the fact that a few fin-de-siécle physicians in Europe and North America thought of themselves as nerve specialists,^3^ it has not always been clear whether many of the earlier figures in the history of neurology practiced as exclusive specialists or thought of their work as distinctly neurological.^4^ As is well known, the boundaries between psychiatric and neurological diseases and their treatments were more amorphous throughout the late nineteenth and early twentieth centuries than current historical literature recognizes,^5^ and many of the physicians[Other P-647] concerned sought to construct practice and research on the nervous system in an integrative fashion, embracing developments in such diverse fields as physiology, pathology, or psychiatry.^6^ Social scientists have claimed that such integrative ideals functioned as encroachments into the dynamic professional terrains of psychology, psychiatry, and even the ecclesiastic professions, a point that William Bynum amplified in his earlier work on neurology in nineteenth- and early twentieth-century Britain.^7^

The predominant view outside of the history of neurology nevertheless remains much as Ellen Dwyer lamented at the close of the twentieth century: "little has been produced on the years since 1918. Although neurology, neuropsychiatry, and neuroscience usually are mentioned in the fast growing historical literature on psychiatry in the twentieth century, they most often *appear in relation* to psychiatry. Few histories start with neurology and then move out to explore its connections with other medical specialties . . ."^8^ Part of the challenge to telling this fuller story has been the general lack of historical literature focusing on neurology's institutional contexts, especially in the period following the First World War. Similarly, the paucity of studies analyzing neurology's international contexts comparatively has made it difficult to appreciate how the field's professional identity emerged cross-culturally.^9^ Thus although intellectual[Other P-648] historians have recorded the diffusion of "neuroscientific concepts" through the multilingual medical research literature,^10^ few institutional, social, or cultural historians have followed suit by exploring variations in national practices and conditions, or cross-cultural connections.^11^ This is surprising, especially for Anglophonic nations like Australia, Canada, Great Britain, New Zealand, and the United States, where there are no language barriers preventing such an analysis, and where it is well understood that many of the field's most significant practitioners crossed national boundaries with startling frequency.^12^ For example, a recent study of Derek Denny-Brown (1901–81, born in New Zealand, trained in London, and appointed director of the neurological unit at Boston City Hospital in 1941) ignores the specific ways in which international experiences and training, not to mention institutional contexts, mediated his clinical outlook, practice, and research.^13^

In this paper, I address these criticisms specifically, choosing for my case the efforts to create a department of neurology at Johns Hopkins Medical School, mainly between 1917 and 1942. It was during this period that Lewis H. Weed (1886–1952), head of the anatomy department and later director of the medical school, established a committee charged with building an internationally renowned department. These efforts foundered, raising several salient points about the professional status of neurology in North America and elsewhere, as well as highlighting the role that international discourses played in defining specifically local understanding of neurology's scope. The institutional papers and personal correspondence used in this study support the view that the connectivity[Other P-649] and conjunctures between diverse international contexts were the integral justification for the Hopkins committee's efforts. Yet, it is clear that local practices and perspectives at Hopkins were predicated upon a supranational ideal of neurology, which was still in the process of forming in the interwar period. What knit together this otherwise locally negotiated ideal were common social and cultural practices and assumptions. Although varying in their local manifestations, these practices and assumptions eventually defined neurology's pedagogy, practicality, and purpose in the years following the Second World War.^14^

## Medical Specialization at Johns Hopkins

Almost since its foundation, Johns Hopkins Hospital and its Medical School—established in 1889 and 1893, respectively—have occupied a privileged place in the story of modern American medicine.^15^ That the ascendance of Johns Hopkins transformed American medical schools and medicine in the twentieth century is a now commonplace view.^16^ In brief, its domination can be attributed to its role in attracting exciting, scientifically oriented medical teachers, who pioneered a curriculum modeled upon mainly German lines, combining practical clinical experience[Other P-650] with extensive training in laboratory practices.^17^ Such famous educators as William Osler (1849–1919), William Stewart Halsted (1852–1922), and William Henry Welch (1850–1934) were celebrated later for teaching their students the arduous art of medicine and the empirical finesse of the scientific method. They emphasized, moreover, how these skills could fit together into an interlocking paradigm of medical practice. The outcomes were a revolution in diagnosis and hospital administration, as well as the discovery of multiple treatments and palliative approaches.^18^

Because of the success of the Johns Hopkins approach, the laboratory assumed a prominent place in medical practice in America, especially in aiding diagnosis and surgical intervention.^19^ Scientific disciplines like physiology and bacteriology, which had hitherto assumed a less significant role in medical school curricula, garnered increasing attention from medical students and physicians alike.^20^ The emergence of these sciences, their subsequent professionalization, and their advocacy for experimental approaches, catalyzed the formation of many medical specialties, especially at Johns Hopkins.^21^ A similar situation occurred in general surgery. Harvey Cushing (1869–1939), for example, following earlier work by William Macewen (1848–1924), Rickman Godlee (1849–1925), and Victor Horsley (1857–1916) in Britain, fashioned the discipline of neurosurgery by improving many of his techniques and knowledge through experiments that he initially conducted on animals or cadavers in Johns Hopkins laboratories and mortuaries.^22^

Like many of his older contemporaries in medicine and surgery, the younger Cushing nevertheless perceived dangers in overspecialization.^23^ His colleague, Adolf Meyer (1866–1950), the psychiatrist and director of the Henry Phipps Psychiatric Clinic at Johns Hopkins, typified the anxieties specialization created when he wrote in 1913 to William Osler, who was by then regius professor of medicine at Oxford, "there must be a liberal willingness to let fields overlap and to have them in common, with frequent consultations and collaboration. Neurasthenia and hysteria and aphasia and apraxia are essential chapters in internal medicine, in neurology and in psychopathology alike."^24^ Osler, no less generalist in his appreciations, was likely sympathetic to Meyer's comment. He had cautioned his students to practice pluralism in their studies and to avoid coveting the "speedy success which often comes from the cultivation of a specialty."^25^

Increasing specialization brought other problems as well. Maintaining any program of medical research in America was an expensive proposition, and economic support was scarce.^26^ Even an institution like Hopkins, idolized in Abraham Flexner's famous 1910 report on medical education, sometimes struggled.^27^ Complaining, for example, of the lack of funds for his research, Adolf Meyer, appointed professor in 1908 and director of the Phipps Psychiatric Clinic in 1913, wrote to Simon Flexner, "I have not had a cent of support for the Psychological Laboratory and only for the last two years about $600 for the Neurological Laboratory. And we are supposed to compete with . . . Imperial German Psychiatry . . . ?"^28^

Fostering emerging medical specialties became one of the many administrative challenges confronting Johns Hopkins. In some cases one worker represented de facto an entire department. Henry M. Thomas (1861– 1925), for example, was the sole practicing specialist in nervous diseases on the Hopkins faculty from 1889 until 1925.^29^ Thomas, as well as Adolf Meyer, conducted neurological research, while Harvey Cushing and his later successor and former clinical assistant Walter Dandy (1886–1946), a pioneer of ventriculography, engaged in experimental neurological operations. ^30^ With such a pedigree, it might seem now that the establishment of a department of neurology would have been an unproblematic development. That it was not is intriguing and informative about the professional status of neurology, nationally and internationally.^31^

## Working Toward a Department of Neurology at Johns Hopkins

Though Hopkins had hired Henry Thomas as a neurological specialist in 1889, the medical school and university struggled for decades to establish a formal department. The result of multiple endeavors was that from the late 1920s until 1969, when a department of neurology was finally established, only a small subdepartment existed in the hospital and university. ^32^ Neurological patients were cared for in the general medical service[Other P-653] and the chronic wards of the hospital.^33^ The teaching of neurology was broken up across many departments, and after Thomas died in 1925, it was reported that there was no one specializing in the subject.^34^ Why was a specialist institutional identity for neurology problematic when other specialties, for example, psychiatry (1909), pediatrics (1912), and ophthalmology (1925), were readily accommodated?

During this period numerous European postgraduates with interests in neurology and psychiatry toured through Hopkins, working for short durations in Meyer's psychiatric clinic and then returning home. Figures from Britain, such as neurologists^35^ Gordon Holmes (1876–1966) and Charles Symonds (1890–1979), pathologist Dorothy Russell (1895–1983), and psychiatrists like David Kennedy Henderson (1884 –1965) and Richard Gillespie (1897–1945), trained for as little as a few weeks to as long as one year in the Phipps Laboratories.^36^ All returned to Britain, improving Britain's impressive international neurological and psychiatric reputation. Even as Hopkins was attracting prominent figures in neurology and psychiatry, it was not retaining them.

The man spearheading the effort to institutionalize neurology at Johns Hopkins was Lewis H. Weed, a former student of Harvey Cushing. Appointed head of the Department of Anatomy at Hopkins Medical School in 1919, he had become dean of the medical school in 1923 and then director in 1929—a post from which he retired in 1946.^37^ His early[Other P-654] research interests were in neuroanatomy and neurophysiology, especially in the biochemical composition of cerebrospinal fluid and its production and circulation through the cerebral ventricles. Like many of his contemporaries, he felt that laboratory analysis of cerebrospinal fluid would improve the diagnosis of neurological diseases such as multiple sclerosis, an insidious neurodegenerative disease.^38^ Although Weed is something of a neglected figure today, he had been a trustee of the Institute for Advanced Study and the Carnegie Institution in Washington, D.C.,^39^ worked tirelessly as chairman of the National Research Council's Medical Sciences Division from 1939 until 1943, and, during that tenure, established an interagency collaboration with the British Medical Research Council.^40^ It was this international collaboration that supported research work on penicillin during the Second World War.^41^

Weed's efforts to build a department of neurology at Hopkins were no less driven. He was a friend of Alan Gregg (1890–1957), the Rockefeller Foundation's medical officer, and by 1931 the holder of the Rockefeller purse for medical research. This friendship with Gregg, however, resulted in little more than cool support for Weed's ambitious plans; it was only in the late 1930s that the Rockefeller Foundation supported Hopkins neurology, and even then its generosity was subdued in comparison with contemporary allotments for neurology in Britain and Canada.^42^

The reasons for this limited assistance are difficult to comprehend. Partly, the dramatic impact of the economic crisis of 1929 stifled promises by other interested philanthropies, such as the Commonwealth Fund, but there were other reasons as well. The plans for neurology at Hopkins were ambitious and unprecedented in the United States. At the time, the Neurological Institute of New York, which had been founded in 1909, had just affiliated in 1925 with Columbia University and the Columbia-Presbyterian[Other P-655] Medical Center.^43^ Likewise, Stanley Cobb (1887–1968) had been given $350,000 from the General Education Board to create a university linked department of neurology at Harvard. Weed, however, judged Cobb's program to be too limited: "Abraham Flexner told me that he was delighted to have a man like Cobb, who was content with ten beds; I do not believe Cobb could develop a real School of Neurology under these conditions. There is no worry of any possible competition about a real department of neurology such as we hope for here. Cobb is a good man, but he is not going to have a great school of neurology."^44^

Weed must have begun deliberating on the subject of neurology sometime in 1918, when Harvey Cushing wrote to him detailing his vision for a national institute for the investigation of disorders of the nervous system, which Cushing had proposed to Abraham Flexner as early as 1917. Cushing hoped to recruit Weed and others, including physiologist Charles Sherrington (1857–1952), to this new institute, where a national school of neurology would be built to rival "the National Hospital for Nervous Diseases in London, or the Salpêtriére in Paris."^45^ But Cushing's project was never realized. At the time, Flexner did not believe that there were "sufficient profitable problems" in neurology for research, and John Fulton later blamed the project's lapse on a mixture of Cushing's stormy personality and the fact that neurology was simply "not a branch of medicine which the philanthropic foundations were at the time disposed to support."^46^

If Cushing's vision for the new institute was the fillip launching Weed's future endeavors at Hopkins, yet another stimulus was at hand by 1920, when he received a draft paper on the organization of an ideal neurological institute from Ernest Spiegel (1895–1985), an Austrian physician who eventually became professor of experimental neurology at Temple[Other P-656] University in 1931.^47^ In his outline, Spiegel summarized the challenges to promoting neurology, captured the peculiar theoretical nature of neurological investigations and constructs, and offered important justifications for supporting such research. Fundamentally, he argued, there was no more important step in "scientific medicine" than the invention of an exact diagnosis, and this was especially true in clinical neurology.^48^ Studies of normal and pathological anatomy, and histology, were important, but more important still were experimental studies localizing functions to specific areas of the nervous system, for these offered the possibility of direct surgical interventions. Spiegel claimed that experimental neurology, if supported adequately, would eventually lay the foundations for many an "experimental therapy of nervous diseases."^49^ He concluded with an inventory of requisite facilities and equipment required for such experimental studies and remarked: "The problems which are to be studied in a neurological institute have to take their origin in clinical observation and so its work must be two-fold: at bedside and in laboratory."^50^ In other words, for clinical neurology to develop adequately, an environment like that offered at Hopkins was the minimum requirement.

Modified aspects of Spiegel's proposal found their way into a 1921 committee report on the teaching of neurology at Hopkins.^51^ Although some neurology was being taught as part of the medical school curriculum,^52^ the committee found that there was "no correlation between the various departments in regard to this required work," and sometimes "the neurological aspects of the general subject" were entirely ignored.^53^ In contrast with the ideal situation Spiegel had outlined, the relationships among[Other P-657] pathology, anatomy, histology, and clinical medicine of Hopkins were not suited to coordinating experimental studies in neurology or even for teaching medical students; medical students with interests in neurology were not training in an environment combining bedside medicine with equivalent laboratory experiences, and such coordination would require the appointment of both a neurological intern and an assistant neurologist. This assistant neurologist was to hold a position in the medical school as an associate professor of neurology.^54^ In addition, this new position would create stronger integration between neurology and psychiatry in the hospital and medical school by centralizing a patient population in wards that could be used by both divisions. Moreover, it would strengthen the research capacity of the Henry Phipps Psychiatric Clinic's neurology laboratory.^55^

Attracting the right person for this position was by no means a simple task. Many European physicians had passed through the Phipps Psychiatric Clinic, and some were natural candidates for recruitment. Adolf Meyer began to sound out former pupils.^56^ He hinted at the possibility in a letter to Charles Symonds (1890–1979), who was then physician of nervous diseases at Guy's Hospital and a former Radcliffe traveling scholar at the Phipps.^57^ But Lewis Weed orchestrated an alternative scheme. In late June 1924, Francis Rouse Martin Walshe (1885–1973), the newly appointed University College Hospital professor of neurology, received the enticing invitation to be visiting professor in neurology in 1925:58 "For some time we have been anxious to invite a neurologist to spend a month or two in Baltimore to give a series of clinics and lectures and advise us concerning the problems in the future development of the neurological clinic of the Johns Hopkins Medical School and Hospital."^59^ Walshe[Other P-658] accepted the position gladly, expressing only joking trepidation at the thought of Baltimore's cold weather.^60^ Later he warned Weed modestly, "I am looking forward eagerly to my stay with you, but shall suffer from 'stage fright' if I think that you expect too much knowledge to distil from my tired brain."^61^

No record indicating exactly why the committee selected Walshe has been found. He was clearly a young, prominent representative of the British clinical tradition in neurology, which by the close of the First World War was world renowned.^62^ London was the chief center of neurological activity in Britain, although Edinburgh and Manchester had prominent workers as well. Especially important was a small specialist hospital located in Bloomsbury, London, which in 1926 was called The National Hospital for the Relief and Cure of Diseases of the Nervous System including Paralysis and Epilepsy.^63^

Despite the National Hospital's international reputation in neurology, Lewis Weed and others on the neurology committee may not have fully appreciated the professional status of neurology in British medicine and the mentality this status engendered. One noteworthy cultural difference, for example, was how British physicians—including many at the National Hospital—viewed medical specialization.^64^ Whereas interwar physicians in the United States rarely denigrated medical specialization, British clinical tradition still advocated a general medical outlook in the 1920s. Archibald Garrod, consulting physician to St. Bartholomew's Hospital, for one, captured this mindset when he acknowledged in 1919:

No more beautiful examples of scientific methods and reasoning can be quoted than those employed by the neurologist in localization of lesions of the brain and spinal cord, and in gaining insight into their nature. It is true that his conclusions are based upon anatomical and physiological observation, which enable him to carry in his mind a stereoscopic picture of the brain as[Other P-659] transparent as the stereoscopic images of the radiographer, but the men who made those observations were, until recently, engaged in the practice of medicine or surgery, and some of them might even have been classed as "popular physicians."^65^

Though this ethos of "generalism" was diminishing in the interwar period, the mentality remained forcefully alive, especially in hospital administrative consciousness. As late as 1932, Garrod's colleague and professor of medicine Francis Fraser (1885–1964)^66^ described a hopeless situation to Alan Gregg at the Rockefeller Foundation: "The plans for a neurological department failed to mature last year and will fail again this year . . . the formation of new special departments does not meet with approval. It is feared that such special departments must in the end mean more specialised instruction for undergraduates with further cramping of the curriculum."^67^ Thus, many British physicians with special interest and training in neurology, some of whom were viewed abroad as leading figures in this new area, held general appointments in hospitals and even self-identified as general physicians. If they consulted as specialists, they did so only in outpatient clinics and private practice.

Francis Walshe was at Hopkins for six weeks, and during his stay he lectured to students, facilitated practical study in the dispensary, and assisted physicians in the treatment of difficult nervous and mental patients.^68^ After Walshe returned to London, Lewis Weed invited him to return to Hopkins as professor of neurology and neurologist-in-chief at the hospital: "As you know, the present facilities for the teaching of Neurology and for the care of patients suffering from neurological disease are hopelessly inadequate. The Advisory Board feels that it is imperative that the general subject of Neurology be developed as rapidly as possible and that Neurology be given its proper place among the medical sciences."^69^ Walshe, however, was not tempted. Citing what seem to be genuine concerns for elderly parents,[Other P-660] family considerations, and patriotism that he had conveyed in his formal letter declining the position,^70^ he nonetheless admitted privately to Weed that the refusal "has not been an easy one by any means, and I hate to make it. However, I know that being what I am, and having thought about it for the past month with much heart searching, it is the right one. I feel that I am doing the second best thing in staying here, but if I came to Baltimore it would have to be without any regrets or looking back over my shoulder if I am to do my duty by Johns Hopkins."^71^

Walshe was divided over whether he had made the right decision. But it may have been an easier decision to make given the obvious challenges he would have confronted in building the department. Although Weed had promised the highest level of administrative cooperation, he could only cite "informal assurance that one of the large foundations will contribute generously."^72^ These informal assurances, even if they materialized, would leave an overwhelming gap to be filled financially, and the future of a neurological department was by no means secured. Comfortable in prestigious positions at both University College Hospital and the National Hospital, Queen Square, Walshe would have been sacrificing a lucrative private practice and university rank for a promising but nonetheless risky role as head of a department yet to be built. For Walshe, the challenge was not very tempting. Yet, as he reminded Weed, "there are as good fish in the sea as ever came out, and I hope you may find whom you want," and certainly the faculty at Hopkins had every reason to feel enthusiastic, even if they were disappointed by Walshe's refusal.^73^ Weed, who had confessed to Walshe that his "own interest in certain aspects of Neurology made me personally feel the overwhelming importance of a good Neurological Institute here," continued to plan his bold project.^74^

In an open proposal written to potential philanthropists (although mainly for the Commonwealth Fund and the Rockefeller Foundation), Weed resorted to a familiar tactic. Describing neurology as the field of medicine that studies the brain, spine, and peripheral nerves, he claimed[Other P-661] it was "no narrow specialty but a wide-spread medical science finding its sphere of activity in many of the common afflictions affecting mankind."^75^ Neurology offered fascinating research questions requiring patience, diligence, and resources; "in this way alone can advances be made." Then, noting the profound possibilities in neurology, Weed argued that Europeans had long recognized neurology's importance:

Great clinics devoted to the study of patients suffering from disease of the nervous system, with well-equipped laboratories for research in this subject, were established over a generation ago; in these clinics, schools of Neurology were developed and in them physicians and students trained. These clinics or institutes were all coordinated with the activities of universities, for without contact with physicists, chemists, biologists and others working in the fundamental sciences, advances in learning in this field could hardly be achieved.^76^

Especially impressive, according to Weed, were the English and French schools, which could claim to have trained most Americans then practicing neurology. The situation was much worse in America, where

the subject of Neurology has never had its proper place. . . . A very few special hospitals devoted to disease of the nervous system (and usually also to Psychiatry) have been founded in different parts of the country but none of these special hospitals is properly coordinated with the activities of a good medical school. . . . As a result the teaching of Neurology in American medical schools has usually been of an inferior type.^77^

Though it is unlikely that he realized it, Weed's positive characterization of especially French and British neurology misrepresented the specialty's professional status—it was not so monolithic.^78^ The contention was, moreover, a bold move in a proposal suggesting that an enormous endowment[Other P-662] of $2,750,000 was required to build such a program in Baltimore.^79^ At a time when philanthropic foundations were investing in research and universities throughout the world, overstating the prowess of European centers risked heightening the competition's profile.^80^

But mentioning the successful development of neurology in Europe also had advantages. The faculty at Hopkins needed to convince skeptical supporters that they merited support, would be able to deliver key ingredients for their program, and furthermore, that neurological practice offered something not provided by general medicine. The existence of purportedly prominent programs in Europe legitimated local calls for a program in neurological research and practice. By 1928, the committee at Hopkins had succeeded in securing funds. The Commonwealth Fund indicated its support for the project, with the caveat that the committee first find a prominent neurologist to head the new department.^81^

As before, this proved surprisingly difficult. Although Hopkins brought in other European figures as visiting professors in neurology between 1929 and 1930, not one proved interested in committing to the position. Early in 1930, the internationally renowned British neurologist Gordon Morgan Holmes (1876–1966) was invited to Baltimore for the autumn term.^82^ The news of Holmes's visit passed through the Rockefeller Foundation, where it was heard, for example, that "Holmes may be possible head for projected institute of neurology at JHU."^83^ Optimistically, the Committee on Neurology recommended "that a definite proposal for the establishment of the Neurological Clinic . . . be prepared for submission to one of[Other P-663] the great foundations."^84^ Holmes was offered the position toward the end of his stay in Baltimore but unexpectedly turned it down.^85^ Why exactly is unknown, but it seems likely that his material circumstances in London played a role. Holmes had a private practice and prominent hospital positions that rivaled those of his former pupil, Francis Walshe.^86^

Holmes's refusal was a distressing blow and caused a period of reflection at Johns Hopkins on the outlook for the entire endeavor. A younger physician, Georges Schaltenbrand (1897–1979), was invited to come from Germany to Baltimore to discuss the project in June of 1931.^87^ With Schaltenbrand's guidance, the Committee on Neurology subsequently drafted a new, definitive statement highlighting eight problems requiring immediate research in neurology. These included the etiology of multiple sclerosis; the relationship between multiple sclerosis and myelopathic disorders; the identification and study of possible viruses causing neuritis, epidemic encephalitis, and poliomyelitis; and the question of whether there were "racial differences in the susceptibility to certain diseases of the nervous system."^88^ This new statement reversed the former contention about the status of neurology in Europe, suggesting instead that neurology throughout the world needed to emulate the ophthalmological and dermatological clinics already in existence. Taking a new line on neurology's international status (one echoed a few months later at the 1931 First International Neurological Congress in Berne), the committee argued that "so far most of the progress in neurology has been due to the efforts of practitioners, internists, and psychiatrists who got interested in the field. The development of some well organized neurological institutes[Other P-664] in all the civilized nations of the world, would promise a great advance beyond the present condition."^89^ In contrast with their previous proposals, this newest formula proposed an evolutionary approach, advocating the "gradual development of the neurological clinic." It maintained "the order of development ought to be, e.g., Out-patient department, Clinical Service, Serological and diagnostic units, Pathology and histopathology, Animal divisions, Special clinics, X-ray and Neurosurgery."^90^

Schaltenbrand had demonstrated great competence and confidence, and like they had of Walshe and Holmes before him, the faculty deemed that he "made a thoroughly happy impression, and his ability to work with other people seemed extraordinarily good—a characteristic which is eminently needed in a subject like clinical neurology."^91^ Weed thus offered him the position of head, and it seemed certain that Schalten-brand would accept. Writing to his friend Alan Gregg, Weed hinted "from our standpoint this action of course means, that if Schaltenbrand accepts, we are prepared to go ahead with the project whenever you are ready to present an official appeal . . . we shall be prepared to go ahead whenever you are ready. If you care to proceed immediately, I can prepare all the necessary official requests."^92^

Weed would be disappointed, but not, as it turned out, by the Rockefeller Foundation. Instead, the Commonwealth Fund, which had promised funds in 1928, now jettisoned support. Writing in his diary, Alan Gregg noted Weed's disappointment.^93^ In a later conversation with an officer from the Commonwealth Fund, Gregg learned that the lack of inclination stemmed both from the ongoing depression, and the fact that preparations had taken so long at Hopkins since they had sent the original proposal. Since the 1928 promise, the Commonwealth Fund's overall funding focus had changed.^94^

The administration at Johns Hopkins had been too slow. Such a situation was made further frustrating by the fact that Schaltenbrand had accepted the position. With a proposal in excess of $6,000,000 and the withdrawal of the main philanthropic player, the situation appeared hopeless:[Other P-665] "They have no hopes for funds at this time."^95^ By April 1932, Lewis Weed had abandoned the project, "reason being insuperable difficulty of securing adequate funds for this development."^96^ Schaltenbrand, seeing that a position at Hopkins had evaporated, apparently left for London.

Ironically, Alan Gregg told Weed that he was relieved at the decision because "it seems a very difficult time to undertake so large a project."^97^ Hopkins's project for neurology—at the scale Weed had envisioned—was a lost cause. What Gregg had not admitted to Weed was that the Rockefeller Foundation was by then negotiating an endowment for the National Hospital in London.^98^ The British neurologists writing the proposal were none other than Gordon Holmes and Francis Walshe—two neurologists whose international prominence had been enhanced by Johns Hopkins's attempts to recruit them as department heads.^99^ Although the National Hospital's funding would finally materialize only in 1935, the Rockefeller support for their proposal was practically guaranteed from the initiation of the project in 1932, and Rockefeller's support for British neurology would be vast in comparison to what they committed to Hopkins in the remaining years of the 1930s and the 1940s.^100^

From April 1932 until December 1934 there were few further improvements in the Hopkins strategy. Although news of the new British developments in neurology undoubtedly reached their ears, no evidence indicates how Hopkins received that information. Letters from Adolf Meyer in 1931 indicate that he was at least supportive of philanthropic contributions to[Other P-666] British psychiatry.^101^ It is likely that such magnanimity would also have existed at Hopkins for neurologists like Holmes and Walshe. Philanthropy, while a zero-sum game for the institutions collectively, was nonetheless facilitating progress in neurology somewhere—just not at Hopkins.^102^

In 1935, the Rockefeller Foundation once again refused Hopkins a small grant of $125,000 allotted over five years, although the reason this time was that their support at Hopkins for other projects was already too extensive.^103^ By 1936 they were more receptive, but only for a small grant to support the salaries of Frank Ford (1892–1970), a long-term member of the Hopkins staff but a newly appointed associate professor in neurology, and Orthello Langworthy, a fellow working in the Hopkins neuro-logical dispensary.^104^ By March of that year, Hopkins had received a grant of $8,000 annually for four years.^105^ Langworthy and Weed submitted a further proposal in 1937 for developing the by then established subdepartment of neurology into an autonomous department.^106^ Despite reading "one of the clearest and most useful" memorandums he had seen, Alan Gregg admitted unhappily that no further support could be forthcoming in the near future, although "you would be right in inferring that we are none the less interested in aiding neurology at Hopkins at a later time."^107^ Gregg did not continue to view the situation so favorably. In 1940 he advocated renewing the Foundation's commitment to Ford's and Langworthy's[Other P-667] salaries because they "have been working under a real handicap in point of instruments," but suggested that a sliding reduction in the amount the Foundation contributed to those salaries each year was in order. He admitted to Weed that this derived from his conviction "that it is reasonable to expect after five years some increasing measure of local support as an evidence of the school's estimation of the value of the services of the group in neurology in teaching and consultation services."^108^ Neurology needed to demonstrate local support. This would be the last grant from the Rockefeller Foundation supporting neurology at Hopkins.^109^ For his part, Weed was grateful: "May I tell you of the great appreciation which we feel toward this renewal of the grant in support of our developing department of neurology. The taper in the appropriation will be very difficult to meet because of many other demands upon the resources of the school here but I can assure you that attempts will be made to carry on the work in neurology at a very high level of efficiency."^110^ Weed was reassuring Gregg that efforts for neurology at Hopkins would continue, but they both knew that the institution could no longer rely upon the Foundation's support. Weed's personal resources for continuing his efforts were probably drying up as well. He had been an advocate for neurology at Hopkins since 1919, and by 1940, faced with increasing responsibilities under wartime pressures at the National Research Council, he was probably unable to continue sustaining his time and energy. Weed's role in the development of neurology at Hopkins was also over.

## Conclusion

Neurology remained a subdepartment at Johns Hopkins until 1969, when the department finally opened. Thomas Turner (1902–2002), dean of the medical faculty at Hopkins, wrote that neurology's status as a division of medicine came under scrutiny again in 1966, and at that time a department of neurology was recommended, with space allocated in the Traylor Building and beds earmarked in the hospital.^111^

By then the landscape of the neurological sciences had shifted firmly toward an integrative biochemical, pathological, and histological[Other P-668] approach, which, when augmented by electroencephalography, the electron microscope, and new clinical scanning technologies, was redefining normal and pathological events in the nervous system. Increasingly, the old divisions among anatomy, pathology, and physiology, as well as between clinical medicine and surgery, were breaking down.^112^ Throughout the world the trend seemed increasingly to be that clinicians might work in preclinical departments just as preclinical scientists might appear in clinical departments.^113^ The division of labor in American medicine was changing in favor of departmental synergy, a point that the professor of neurology at Columbia University, H. Houston Merritt (1902–79), was already making in 1951 when he told freshman medical students, "The old barriers have been destroyed . . . it is now the aim of the Medical School to give the students an integrated knowledge of the human body and its reaction to disease and injury."^114^

It is clear that in the 1920s, a preconception of the supremacy of the European medical tradition continued to influence American medicine. In part, however, that preconception was rhetorical. In this case, it allowed administrators and physicians at Johns Hopkins to argue for modernization on the grounds that their status quo was inadequate or inefficient. Modernization seemingly called for a socio-institutional transformation only; progress was deemed to derive from the further incorporation of a division of labor into the structure of hospital medicine and medical training.

Contemporaneously, young European physicians and scientists in the early stages of their careers (as well as some of the more established medical elite) were falling under the sway of a new model of laboratory medicine that was being nurtured by American philanthropy.^115^ Thus, while figures at Johns Hopkins and elsewhere in North America were viewing the numerous European brain institutes and teaching hospitals devoted to nervous diseases as evidence of a need to incorporate specialist neurology into American medical practice and curriculum, physicians and scientists with "modernist" inclinations in Britain and Europe were reconsidering[Other P-669] the role of the laboratory in medicine and neurology. Perhaps the physicians at the National Hospital, Queen Square were successful in acquiring Rockefeller Foundation support because they were seeking to adopt this newest form of medical practice. In contrast, their competitors in Baltimore were arguing that they needed financial capital to merely build specialist departments.

More broadly, it is noteworthy that in the post progressive-era United States, the Neurology Committee at Hopkins cited European and British centers of neurology as exemplars of how neurology could be developed in Baltimore and, moreover, that they sought leading figures mainly from Britain to head their new department. Although they might have used a North American tradition of neurology in their appeals for philanthropic aid, Lewis Weed and others turned instead to an internationalist mantra. In a period of Atlantic crossings and progressive-minded internationalist discourses, neurology's purpose and practicality became located within a supranational consensus forged between physicians and administrators with similar appreciations and dispositions.

Within this framework, then, was both a degree of unreality and competition on an international scale. Its unreality had to do with the deployment of a language that often failed to grapple with the nuances of local realities. Thus, although the clinical tradition of neurology was described as preeminent in Britain, many of the pivotal figures belonging to that tradition had little more institutional or research support than did their colleagues in North America. Hence, when Gordon Holmes died in 1965, the author of his *London Times* obituary could describe him in these heroic terms:

It is of interest to put on the record, in the highly organised medical world of today, that the principal scene of Holmes' labours was a small voluntary hospital, which though for many years a world-famous school of neurology, was neither recognised nor supported by a university. His very numerous additions to medical knowledge were the hard-won fruit of the unsubsidised labour of the spare time of a physician who was also engaged in and dependent upon private practice. This of course had been equally true of his predecessors in the National Hospital: Jackson, Ferrier, Bastian, Gowers, and Horsley—some of the greatest names in modern neurology—and Holmes was perhaps the last of this remarkable group of men; all inexhaustible, forceful, and immensely able, who created the prestige of British neurology out of their own intellectual resources.^116^

How had a world-famous school of neurology prevailed under such conditions? In the epistolary economy of the interwar period, reports of the local professional status of neurology trafficked in both directions across the Atlantic. Rife with comparisons, the authors of these messages—which existed as letters, reports, journal articles, or even personal conversation— routinely claimed that conditions elsewhere were better.^117^ These messages were negotiated and operated to fashion a supranational ideal for neurology's professional identity, which was informing higher level administrative decision making and defining the field's purpose and practicality. In part, for example, the National Hospital's fame was generated by its international acclaim in the Hopkins Neurology Committee's reports, which were circulated to the Rockefeller Foundation and others. At the least, from the vantage point of an international philanthropic organization, the depictions of the National Hospital, Queen Square as the exemplary model for neurology and an environment resplendent with sought-after figures made the hospital an attractive institution for support. Queen Square reaped substantial benefits, including a lavish endowment in 1935 for research. In contrast, Johns Hopkins received only small support until the late 1960s.[Other P-671]

## Footnotes

1 This excellent literature includes: GuthrieLeonardContributions to the Study of Precocity in Children, The History of Neurology: the Fitzpatrick Lectures on the History of Medicine Delivered at the Royal College of Physicians in the Years 1907, 1908LondonEric G. Millar1921GarrisonFielding H.History of Neurology in DanaCharles L.Text-book of Nervous Diseases for the Use of Students and Practitioners of Medicine10th ed.New YorkWilliam Wood1925 pp. xv–lviGreenblattSamuelThe Major Influences on the Early Life and Works of John Hughlings JacksonBull. Hist. Med.196539346–3765318128ClarkeEdwinO'MalleyD. D.The Human Brain and Spinal CordBerkeleyUniversity of California Press1968McHenryLawrenceGarrison's History of NeurologySpringfield, Ill.Charles C. Thomas1969TemkinOwseiThe Falling Sickness: A History of Epilepsy From the Greeks to the Beginnings of Modern NeurologyBaltimoreJohns Hopkins Press1971DeJongRussellA History of American NeurologyNew YorkRaven Press1982PorterRoyThe Greatest Benefit to Mankind: A Medical History of HumanityNew YorkW. W. Norton pp. 534–551OchsSidneyA History of Nerve Functions: From Animal Spirits to Molecular MechanismsCambridgeCambridge University Press2004

2
					TalleyCollin L.The Emergence of Multiple Sclerosis, 1870–1950Perspect. Biol. Med.200548385–38710.1353/pbm.2005.007916085995 FarrerasIngrid D.HannawayCarolineHardenVictoria A. eds., Mind, Brain, Body, and Behavior: Foundations of Neuroscience and Behavioral Research at the National Institutes of HealthBiomedical and Health Research vol. 62AmsterdamIOS Press2004
				

3
					HaymakerWebbSchillerFrancis eds. The Founders of Neurology: One Hundred and Forty-six Biographical SketchesSpringfield, Ill.Charles C. Thomas1970 BlusteinBonnie EllenPreserve Your Love For Science: Life of William Hammond, American NeurologistCambridgeCambridge University Press1991 GoetzChristopherBonduelleMichelGelfandTobyCharcot: Constructing NeurologyNew YorkOxford University Press1995
				

4
					BlusteinBonnie EllenNew York Neurologists and the Specialization of American MedicineBull. Hist. Med.197953170–183389317
				

5 Exceptions are CasperStephenThe Idioms of Practice: British Neurology, 1880–1960 (Ph.D. diss., University College London2007BallengerJesse F.Self, Senility, and Alzheimer's Disease in Modern America: A HistoryBaltimoreJohns Hopkins University Press2006 pp. 36–55SmithRogerThe Fontana History of the Human SciencesLondonHarperCollins1997OppenheimJanetShattered Nerves: Doctors, Patients, and Depression in Victorian EnglandNew YorkOxford University Press1991 pp. 40–41 c.f. RousseauGeorge S.Nervous Acts: Essays on Literature, Culture and SensibilityBasingstokePalgrave Macmillan2004

6
					GeisonGeraldMichael Foster and the Cambridge School of Physiology: The Scientific Enterprise in Late Victorian SocietyPrincetonPrinceton University Press1978 LloydJames HendrieThe Neuroses of PeaceArchives Neurol. Psych.192041–7 WalsheFrancisTraining of the NeurologistArch. Neurol. Psych.193329380–91
				

7
					AbbottAndrewThe System of the Professions: An Essay on the Division of Expert LaborChicagoUniversity of Chicago Press1988 on neurology specifically, see pp. 319–323 BynumWilliamThe Nervous Patient in 18th- and 19th-century Britain: The Psychiatric Origins of British Neurology in Lectures on the History of Psychiatry: The Squibb SeriesLondonGaskell1990 pp. 115–127
				

8 The emphasis is mine. DwyerEllenToward New Narratives of Twentieth-Century MedicineBull. Hist. Med.200074786–793 p. 7881120126710.1353/bhm.2000.0176 For representative examples of Dwyer's point, see Brumberg'sJoan JacobsFasting Girls: The History of Anorexia NervosaNew YorkVintage Books2000 and GrobGerald N.Mental Illness and American Society, 1875–1940PrincetonPrinceton University Press1983 pp. 50–62 Historians interested in neurology have begun to examine these issues. See RoseF. CliffordHistoriography: An IntroductionJ. Hist. Neurosci.20021135–371201257410.1076/jhin.11.1.35.9102 and, in the same issue, SöderqvistThomasNeuro-biographies: Writing Lives in the History of Neurology and the Neurosciences pp. 38–4810.1076/jhin.11.1.38.909912012575 and KraghHelgeProblems and Challenges in the Historical Study of the Neurosciences pp. 55–6210.1076/jhin.11.1.55.909712012577

9 Exceptions include: KoehlerPeterThe Evolution of British Neurology in Comparison with Other Countries in A Short History of Neurology: The British Contribution, 1660–1910 ed. RoseF. CliffordOxfordButterworth and Heinemann1999 pp. 58–74SolomonSusan Gross ed., Doing Medicine Together: Germany and Russia Between the WarsTorontoUniversity of Toronto Press2006 pp. 325–368407–461

10
					YoungRobertMind, Brain, and Adaptation in the Nineteenth CenturyNew YorkOxford University Press1970 HarringtonAnneMedicine, Mind, and the Double Brain: A Study in Nineteenth-Century ThoughtPrincetonPrinceton University Press1987 SmithRogerInhibition: History and Meaning in the Sciences of the Brain and MindLondonFree Association Books1992 and JacynaL. StephenLost Words: Narratives of Language and the Brain, 1825–1926PrincetonPrinceton University Press2000

11 Notable exceptions are: WeiszGeorgeDivide and Conquer: A Comparative History of Medical SpecializationOxfordOxford University Press2006 esp. pp. 210–213LanskaDouglasChmuraT. A.GoetzChristopherPart 1: The History of 19th Century Neurology and the American Neurological AssociationAnn. Neurol.200353Suppl. 4S2–S261272208710.1002/ana.8888BarberisDanielaChanging Practices of Commemoration in NeurologyOsiris199914102–1171197129110.1086/649302PressmanJackLast Resort: Psychosurgery and the Limits of MedicineCambridgeCambridge University Press1998

12 For example, see: FraenkelG. J.Hugh Cairns: First Nuffield Professor of Surgery, University of OxfordOxfordOxford University Press2003

13
					VilenskyJoel A.GilmanSidSinishPandy R.Denny-Brown, Boston City Hospital, and the History of American NeurologyPerspect. Biol. Med.200447505–5181546717410.1353/pbm.2004.0074
				

14 A methodological point must be made about such cross-cultural comparison. In many respects, the challenge of producing this type of argument from archival sources is that it forces at once painstaking detail and an understanding of the subtleties of national, local, and connected contexts. At the same time, editorial considerations make it challenging to render the narrative brief and readable. An elegant example showing markedly similar tensions is Mark Harrison, "Disease, Diplomacy, and International Commerce: The Origins of International Sanitary Regulation in the Nineteenth Century," *J. Global Hist.*, 2006, *1*: 197–217. On the role of appreciations and dispositions, see BourdieuPierreHomo AcademicusCambridgePolity Press1992

15 See, for example, LudmererKennethLearning to Heal: The Development of American Medical EducationNew YorkBasic Books1985 pp. 38–4257–5863219–220TurnerThomasHeritage of Excellence: The Johns Hopkins Medical Institutions, 1914 –1947BaltimoreJohns Hopkins University Press1974ChesneyAlanThe Johns Hopkins Hospital and The Johns Hopkins University School of Medicine: A Chronicle2 vols.BaltimoreJohns Hopkins Press1958

16 For a most recent discussion about the influence of Hopkins's model, see PutnamConstanceThe Science We Have Loved and Taught: Dartmouth Medical School's First Two CenturiesHanoverUniversity Press of New England2004 pp. 86–160 see also StarrPaulThe Social Transformation of American MedicineNew YorkBasic Books1982 pp. 115–118StevensRosemaryAmerican Medicine and the Public InterestNew HavenYale University Press1971 pp. 41–4256–5869 For American medicine and philanthropy, see BrownE. RichardRockefeller Medical Men: Medicine and Capitalism in AmericaBerkeleyUniversity of California Press1979

17
					LudmererKennethThe Rise of the Teaching Hospital in AmericaJ. Hist. Med. All. Sci.198338389–41410.1093/jhmas/38.4.3896358338 LudmererThe Plight of Clinical Teaching in AmericaBull. Hist. Med.198357218–2296347290
				

18
					FlexnerSimonFlexnerJames ThomasWilliam H. Welch and the Rise of Modern MedicineBostonLittle, Brown1954 CushingHarveyThe Life of Sir William Osler2 vols.OxfordClarendon Press1925PMC23481716015960
				

19
					WarnerJohn HarleyScience in MedicineOsiris1985137–581162166210.1086/368637 WarnerIdeals of Science and their Discontents in Late Nineteenth-Century American MedicineIsis199182454–478195530010.1086/355837
				

20
					GeisonGeraldDivided We Stand: Physiologists and Clinicians in American Context in The Therapeutic Revolution: Essays in the Social History of American Medicine ed. VogelMorris J.RosenbergCharlesPhiladelphiaUniversity of Pennsylvania Press1979 pp. 115–129
				

21
					Cho KimOck-JooThe Integration of Science with the Healing Art: Harvey Cushing's Development of Neurosurgery, 1896–1912 (Ph.D. diss., University of Minnesota1998
				

22
					GreenblattSamuelHarvey Cushing's Paradigmatic Contribution to Neurosurgery and the Evolution of his Thoughts About SpecializationBull. Hist. Med.200377789–8221465758410.1353/bhm.2003.0168 On Rickman Godlee, see FeilingAnthonyA History of the Maida Vale Hospital for Nervous DiseasesLondonButterworth1958 appendices 1–3

23
					BlissMichaelHarvey Cushing: A Life in SurgeryOxfordOxford University Press2005
				

24 Adolf Meyer to William Osler, 7 April 1913, I/2963/3 Osler, Sir William, The Adolf Meyer Collection, The Alan Mason Chesney Medical Archives of the Johns Hopkins Medical Institutions (hereafter referred to as AMCMA).

25
					CamacC. N. B. ed., Counsels and Ideals From the Writings of William Osler2nd ed.LondonHenry Frowde1906 pp. 182–187 p. 182
				

26
					TurnerHeritage of Excellence (n. 15 pp. 149–175
				

27
					FlexnerAbrahamMedical Education in the United States and Canada: A Report to the Carnegie Foundation for the Advancement of TeachingBostonMerrymont Press1910 See, for example, p. 12: "This was the first medical school in America of genuine university type, with something approaching adequate endowment, well equipped laboratories conducted by modern teachers, devoting themselves unreservedly to medical investigations and instruction, and with its own hospital, in which the training of physicians and the healing of the sick harmoniously combine to the infinite advantage of both."

28 Adolf Meyer to Simon Flexner, 2 October 1919, 1/1187/1 Flexner, Simon, The Adolf Meyer Collection, AMCMA; on Meyer see The Adolf Meyer CollectionRepository Guide to the Personal Papers Collections of Alan Mason Chesney Medical Archives Johns Hopkins Institutions available at http://www.medicalarchives.jhmi.edu/sgml/meyera.html (last accessed 2992004

29 Exact details of Henry M. Thomas's appointment are difficult to locate. In his papers published in the *Johns Hopkins Medical Bulletin*, his title is given routinely as "Clinical Profession of Neurology." For instance, see ThomasH. M.Decussation of the Pyramids—An Historical InquiryJohns Hopkins Med. Bull.191021304–311 on the other hand, Chesney writes that he was appointed as "Clinical Professor of Diseases of the Nervous System": ChesneyThe Johns Hopkins Hospital and The Johns Hopkins University School of Medicine (n. 15 p. 9113472307 There is no corroborating evidence in Thomas's papers. See the files marked: Thomas, Henry M. Sr., Fac. 86–87 (Sch. Med), The Biographical Collection, AMCMA. See also Thomas, Henry M. in The National Cyclopaedia of American Biography: Being the History of the United States as Illustrated in the Lives of the Founders, Builders, and Defenders of the Republic, and of the Men and Women Who are Doing the Work and Moulding of the Thought of the Present Time192920163

30 On Dandy, see Walter Edward DandyJAMA19461301257 see also DandyWalterVentriculography Following the Injection of Air in the Cerebral VentricalsAnn. Surg.1918685–111786394610.1097/00000658-191807000-00002PMC1426769

31
					TurnerHeritage of Excellence (n. 15 pp. 202–208
				

32 This was the condition of neurology in universities and medical colleges throughout America at the time: Barrow Neurological Institute of St. Joseph's HospitalArizonaHorizons in Neurological Education and Research: Commemorative Volume of Dedication Symposium and AddressesSpringfield, Ill.Charles C. Thomas1965 pp. 41–46

33 Lewis Weed to Alan Gregg, 29 October 1937, Correspondence Neurology Committee on Dec. 1934 –May 1940, The Lewis H. Weed Collection, AMCMA [enclosure: *A Plan for the**Development of Neurology at the Johns Hopkins University*].

34 Required Teaching in Neurology, c. 1920, Correspondence Neurology Committee, Sept. 1917–1921, The Lewis H. Weed Collection, AMCMA. A useful discussion of teaching in neurology and psychiatry in America appears in BrandJeanne L.Neurology and Psychiatry in The Education of American Physicians: Historical Essays ed. NumbersRonaldBerkeleyUniversity of California Press1980 pp. 226–249

35 Although I use the term "neurologist" throughout this paper, the term today implies a greater sense of professional identity than existed in neurology in this period. A useful discussion of neurology's definition appears in EadieMervynThe Flowering of a Waratah: The History of Australian Neurology and of the Australian Association of NeurologistsEastleighJohn Libbey; 2000 pp. 3–42 also see KushnerHowardA Cursing Brain: The Histories of Tourette SyndromeCambridgeHarvard University Press1999 p. 235 footnote 21

36 Henderson to Meyer, 29 October 1911, I/1569/1 Henderson, David K; Gordon M. Holmes to Meyer, 2 October 1912 (Holmes had spent a short time there) I/1759/1 Holmes; Symonds to Meyer, 12 June 1923, I/3753/1 Symonds, Charles P. Penfield to Meyer 21 February 1929, I/3046/2 Penfield, Wilder; Hurst to Meyer, 11 March 1920, I/1848/1 Hurst, Arthur F. The Adolf Meyer Collection, AMCMA.

37
					Repository Guide to the Personal Papers Collections of Alan Mason Chesney Medical Archives Johns Hopkins InstitutionsThe Lewis H. Weed Collection available at http://www.medical archives.jhmi.edu/sgml/weed.html (last accessed 2992004
				

38 See the description of these early theories in McAlpineDouglasCompstonNigelLumsdenCharlesMultiple SclerosisLondonE&S Livingston1955 pp. 257–258

39
					GariepyThomasJohn Farquhar Fulton and the History of Science SocietyIsis199990Suppl.): S7–S27 p. S19
				

40 For information on this Anglo-American collaboration, see CasperStephenThe Origins of the Anglo-American Research Alliance and the Incidence of Civilian Neuroses in Second World War BritainMed. Hist200852327–3461864178910.1017/s0025727300002660PMC2448977

41 Edward Mellanby to G. E. Brown, 7 January 1944, FD 1/6579 National Archives, London.

42 See Resolution 40008, 1/19/1940, for an administrative overview, Record Group (hereafter referred to as RG) 1.1, Series 200, box 94, folder 1132: Johns Hopkins Neurology 1930–1935, Rockefeller Archive Center, Sleepy Hollow, New York (hereafter referred to as RAC).

43 Reprint of Official Statement Sent to the Press on 12 March 1925, Announcing Affiliation of the Neurological Institute with the Columbia-Presbyterian Medical Center (CPMC); Neurological Institute of New York Collection, 1917–1983 (bulk 1930–1960), Affiliation with CPMC: Correspondence, Documents, 1925–1955, Neurological Institute, box 1, fo. 1; Columbia University Archives and Special Collections; see also GreenJohnThe Origins of Neurological Institutes in Barrow Neurological InstituteHorizons in Neurological Education and Research (n. 32 pp. 125–199147

44 Lewis Weed to H. J. Seymour, 6 May 1926, Correspondence Neurology Committee March 1924 –Nov 1927, The Lewis H. Weed Collection, AMCMA; on Cobb, see WhiteBenjaminStanley Cobb: A Builder of the Modern NeurosciencesBostonUniversity Press1984

45 Cushing quoted in FultonJohnHarvey Cushing: A BiographySpringfield, Ill.Charles C. Thomas1946 p. 445

46 Ibid.FultonJohnHarvey Cushing: A BiographySpringfield, Ill.Charles C. Thomas1946 p. 445 p. 450.

47
					MeagherRichardBuchheitWilliamNarayanRaj K.The History of Neurosurgery at Temple UniversityNeurosurg.200455688–697 p. 69110.1227/01.neu.0000134559.62469.c115335437 Spiegel's ambitiousness in writing such a proposal is similar to other aspects of his career. He had, for example, published a lengthy review of the progress made in neurology and psychiatry in 1928: SpiegelErnestMonographien aus den Gesamptgebiete der Neurologie und Psychiatric Heft 54Springer1928

48
					SpiegelE.Vienna University—Outline of the Organization of a Neurological Institute pp. 1–4 p. 1 Correspondence Neurology Committee March 1917–Nov 1921, The Lewis H. Weed Collection, AMCMA.

49
					SpiegelE.Vienna University—Outline of the Organization of a Neurological Institute pp. 1–2
				

50
					SpiegelE.Vienna University—Outline of the Organization of a Neurological Institute p. 2
				

51 To the Faculty of the Johns Hopkins Medical School, 17 March 1921, Correspondence Neurology Committee March 1917–Nov 1921, The Lewis H. Weed Collection, AMCMA.

52 *Required Teaching in Neurology and Elective Courses in Neurology*, Correspondence Neurology Committee March 1924 –Nov 1927, The Lewis H. Weed Collection, AMCMA.

53 Committee on Neurology to Faculty of the Johns Hopkins Medical School, 17 March 1921, Correspondence Neurology Committee March 1924 –Nov 1927, The Lewis H. Weed Collection, AMCMA.

54 *Plan for the Organization of the Department of Neurology*, Correspondence Neurology Committee March 1924 –Nov 1927, The Lewis H. Weed Collection, AMCMA.

55 Ibid.

56 Adolf Meyer to Charles Symonds, 29 May 1923, I/3753/1 Symonds, C. P., The Adolf Meyer Collection, AMCMA.

57 Symonds to Meyer, AMCMA (n. 36).

59 Warfield Longcope to Francis Walshe, 24 June 1924, MS ADD 301, Library Services, Walshe Papers University College London (hereafter referred to as UCL).

60 Warfield Longcope to Francis Walshe, 4 September 1924, MS ADD 301, UCL.

61 Francis Walshe to Lewis Weed, 12 December 1924, The Lewis H. Weed Collection, Walshe, F. M. R., 1922–1937, AMCMA.

62
					Anon.Neurology in the WarBMJ19192790–791
				

63 There are several histories of the hospital and its neighborhood. See GowersErnestQueen Square and the National Hospital, 1860–1960LondonEdward Arnold1960HolmesGordonThe National Hospital, Queen Square, 1860–1948EdinburghE&S Livingston1954HamiltonGodfrey HeathcoteQueen Square: Its Neighbourhood & Its InstitutionsLondonL. Parsons1926RawlingsB. BurfordA Hospital in the Making: A History of the National Hospital for the Paralyzed and EpilepticLondonPitman1913

64
					MartinJames PurdonBritish Neurology in the Last Fifty Years: Some Personal ExperiencesProc. R. Soc. Med.1971641055–1059494752710.1177/003591577106401022PMC1812784 MartinReminiscences of Queen SquareBMJ198128363071640–1642679759210.1136/bmj.283.6307.1640PMC1507480
				

65
					GarrodArchibaldThe Laboratory and the Ward in Contributions to Medical and Biological Research, Dedicated to Sir William Osler Bart., M.D., F.R.S. In Honour of his Seventieth Birthday, July 12, 1919 By His Pupils and Co-Workers 2 vols. New YorkPaul B. Hoeber1919 pp. 60–61
				

66
					McMichaelJohnFraser, Sir Francis Richard (1885–1964) in Oxford Dictionary of National Biography ed. MatthewH. C. G.HarrisonBrianOxfordOxford University Press2004 available at http://www.oxforddnb.com/view/article/33251 (last accessed 2912007
				

67 Francis Fraser to Alan Gregg, 6 June 1932, RG 1.1, folder 265, box 20, series 401, RAC.

68 Henry Thomas to Francis Walshe, 17 October 1924, MS ADD 301, UCL.

69 Lewis Weed to Francis Walshe, 31 March 1925, Walshe, F. M. R., 1922–1937, The Lewis H. Weed Collection, AMCMA.

70 Francis Walshe to Lewis Weed, 16 April 1925a, Walshe, F. M. R., 1922–1937, The Lewis H. Weed Collection, AMCMA.

71 Francis Walshe to Lewis Weed, 16 April 1925b, Walshe, F. M. R., 1922–1937, The Lewis H. Weed Collection, AMCMA. Walshe apparently recalled seeing a number of extraordinary neurological cases while at Hopkins. See CritchleyMacdonald1885–1973 Sir Francis Walshe in The Ventricle of Memory: Personal Recollections of Some NeurologistsNew YorkRaven Press1990 pp. 195–206203

72 Weed to Walshe (n. 69), AMCMA.

73 Walshe to Weed (n. 71), AMCMA.

74 Lewis Weed to Francis Walshe, 29 April 1925, Walshe, F. M. R., 1922–1937, The Lewis H. Weed Collection, AMCMA.

75
					A Proposal for a University Clinic and Institute of Neurology at the Johns Hopkins Medical School and Hospital pp. 1–8 p. 1 Correspondence Neurology Committee 31924–111927 The Lewis H. Weed Collection, AMCMA
				

76
					A Proposal for a University Clinic and Institute of Neurology at the Johns Hopkins Medical School and Hospital pp. 2–3
				

77
					A Proposal for a University Clinic and Institute of Neurology at the Johns Hopkins Medical School and Hospital p. 3
				

78 See Norman Dott's enclosures to Harvey Cushing, 26 September 1929, where he says in a proposal to the Rockefeller Foundation, "I think these last considerations apply with special force to Edinburgh, among other British Schools, in which neurology in the wide sense of the term does not occupy quite the definitely recognised status of the special subject that it does in most American and many Continental Schools." Dott, Norman M. 1924 –1938, The Harvey Williams Cushing Papers in the Yale University Library, Manuscripts and Archives, Yale University Library, Microfilms Series I, *box 24*, 448, Microfilm Reel 21; on conditions in France, see BarberisChanging Practices of Commemoration in Neurology (n. 11 pp. 102–10810.1086/64930211971291

79
					A Proposal for a University Clinic and Institute of Neurology at the Johns Hopkins Medical School and Hospital (n. 75 pp. 6–7
				

80
					SchneiderWilliamThe Men Who Followed Flexner: Richard Pearce, Alan Gregg, and the Rockefeller Foundation Medical Divisions, 1919–1951 in Rockefeller Philanthropy and Modern Biomedicine: International Initiatives from World War I to the Cold War ed. SchneiderWilliamBloomingtonIndiana University Press2002 LawrenceChristopherRockefeller Money, the Laboratory and Medicine in Edinburgh, 1919–1930: New Culture in an Old CountryNew YorkUniversity of Rochester Press2005 PenfieldWilderThe Difficult Art of Giving: The Epic of Alan GreggBoston1967
				

81 Lewis Weed to Robert Lambert, 17 June 1930, RG 1.1, Series 200, box 94, folder 1132 Johns Hopkins Neurology 1930–1935, RAC.

82 Harvey Cushing to Gordon Holmes, 22 May 1930, Holmes, Gordon, 1915–1936, The Harvey Williams Cushing Papers in the Yale University Library, Manuscripts and Archives, Yale University Library, Microfilms Series I, box 36, 703, Microfilm Reel 30.

83 Robert Lambert to Daniel O'Brien, 1 May 1930, RG 1.1, series 401, box 20, folder 265, RAC; the RF had learned of Holmes's interest in the position even before he had been officially invited, see Daniel O'Brien Diary, 1 March 1930, no page number provided, RAC.

84
					Report of the Committee on Neurology pp. 1–4 specifically p. 4 Correspondence Neurology Committee Nov. 1927 to Oct. 1930, The Lewis H. Weed Collection, AMCMA.

85 *Report of the Committee to the Advisory Board of the Medical Faculty*, 18 October 1930, Correspondence Neurology Committee Nov. 1927 to Oct. 1930, The Lewis H. Weed Collection, AMCMA. Report of Committee on Neurology, 17 April 1931, Correspondence Neurology Committee Dec. 1930–1934, The Lewis H. Weed Collection, AMCMA.

86 In a 1932 interview with Sir Thomas Lewis (1881–1945), Daniel O'Brien (1894 –1958), the Rockefeller Foundation's European medical sciences officer, learned that several significant figures in Britain considered it a mistake for "American Universities to try to import English and foreign neurologists. It would be best to try to train Americans of promise for considerable period of years in Europe." Daniel O'Brien Diary, 8 March 1932, p. 40, RAC.

87 Report of Committee on Neurology, 17 April 1931 (n. 85). Schaltenbrand had been recommended to Lewis Weed by Gordon Holmes as one of the "most important young men in neurology" in the world. Daniel O'Brien Diary, Visit to Baltimore—28 September 1932, p. 152, RAC.

88
					Problems of Neurology pp. 1–7 p. 2 Report of Committee on Neurology, 17 April 1931, Correspondence Neurology Committee Dec. 1930–1934, The Lewis H. Weed Collection, AMCMA.

89
					Problems of Neurology p. 2 On the First International Neurological Congress in Berne, see Proceedings of the First International Neurological Congress, Berne (Switzerland), August 31 to September 4, 1931BerneStämpfli1932 esp. pp. 375–376

90
					Problems of Neurology (n. 88 p. 7AMCMA
				

91 Lewis Weed to Alan Gregg, 11 June 1931, RG 1.1, Series 200, box 94, folder 1132, Johns Hopkins Neurology 1930–1935, RAC.

92 Ibid.

93 Alan Gregg's Diary, 28 October 1931, RG 1.1, Series 200, box 94, folder 1132 Johns Hopkins Neurology 1930–1935, RAC.

94 Ibid., 18 November 1931.

95 MM's Diary, 3 February 1932, RG 1.1, Series 200, box 94, folder 1132 Johns Hopkins Neurology 1930–1935, RAC.

96 Gregg's Diary, 11 April 1932 (n. 93).

97 Ibid.

98 April 16, 1930 (Oct 27 1937 rec.); 4/10/35 National Hospital for Diseases of the Nervous System—London, RG 1.1, Series 401, box 20, folder 265, A National Hospital for Nervous Diseases, 1930–1933, RAC; see also Minutes of the Rockefeller Foundation104193535109–35112

99 Lambert to O'Brien (n. 83); Lewis Weed to Robert Lambert, 5 November 1930; Lewis Weed to Robert A. Lambert, 12 November 1930; RG 1.1, Series 401, box 20, folder 265, A National Hospital for Nervous Diseases, 1930–1933, RAC.

100 The approximate total contribution between 1930 and 1942 from the Rockefeller Foundation for all of its projects in British neurology, both at the National Hospital and other places, was more than $1,000,000, partly because the British pound was valued at almost four dollars to one pound. Nowhere do their contributions to neurology seem to have been greater, including the Montreal Neurological Institute, although they deemed their overall contribution to mental health comparable to allocations "made or under consideration in Boston, Baltimore, and Chicago." Minutes of the Rockefeller Foundation (n. 981041935 p. 35109

101 Walshe to Meyer, 6 March 1931, I/3947/1 Walshe, F. M. R., The Adolf Meyer Collection, AMCMA.

102 This point is contentious; yet it is worth noting that many of these men believed in the nobility of the practice of medicine and the inherent goodness of medical research. To suggest that they would have been jealous of the accomplishments at other institutions would, I think, misrepresent their views of progress in scientific and medical research.

103 Lewis Weed to Alan Gregg, 10 January 1935 RG 1.1, Series 401, RG 1.1, Series 200, box 94, folder 1132 Johns Hopkins Neurology 1930–1935, RAC; Alan Gregg to Lewis Weed, 31 July 1935; Gregg's Diary, 14 October 1935 (n. 93).

104 On Ford and Langworthy, see Weed to Gregg, 10 January 1935 (n. 103); on Rockefeller receptiveness, see: Lewis Weed to Alan Gregg, 20 December 1935; Alan Gregg to Lewis Weed, 3 March 1936, RG 1.1, Series 200, box 94, folder 1133 Johns Hopkins University— neurology, 1936–1941, RAC. For Ford, see the essays In Memory of Dr. Frank R. FordJohns Hopkins Med. J.1971128100–109

105 Gregg to Weed, 3 March 1936 (n. 104).

106 Orthello Langworthy to Alan Gregg, 29 October 1937 [enclosure: A Plan for the Development of Neurology at the Johns Hopkins University], RG 1.1, Series 200, box 94, folder 1133 Johns Hopkins University—neurology, 1936–1941, RAC. The proposal can also be found in *A Plan for the Development of Neurology at Johns Hopkins University*, Neurology Committee Dec. 1934 –May 1940, The Lewis H. Weed Collection, AMCMA.

107 Alan Gregg to Orthello Langworthy, 7 December 1937, RG 1.1, Series 200, box 94, folder 1133 Johns Hopkins University—neurology, 1936–1941, RAC.

108 Alan Gregg to Lewis Weed, 19 January 1940, RG 1.1, Series 200, box 94, folder 1133 Johns Hopkins University—neurology, 1936–1941, RAC.

109 Ibid.

110 Lewis Weed to Alan Gregg, 20 January 1940, RG 1.1, Series 200, box 94, folder 1133 Johns Hopkins University—neurology, 1936–1941, RAC.

111
					TurnerThomasAccounting of a Stewardship: The Johns Hopkins Medical School, 1957– 1968BaltimoreJohns Hopkins University Press1969 p. 41
				

112 On this point, see FarrerasHannawayHardenMind, Brain, Body, and Behavior (n. 2 pp. 13–67

113 For a similar view, see KeatingPeterCambrosioAlbertoBiomedical Platforms: Realigning the Normal and the Pathological in Late-Twentieth Century MedicineCambridgeMIT Press2003 pp. 17–19

114
					MerrittH. HoustonMedicine in the Last Half of the Twentieth Century Opening Address to Columbia University Medical Students, 17919514 Lectures, 1951–62, Merritt Papers, box 2, fo. 14, Columbia Health Centre Archives.

115
					LawrenceRockefeller Money (n. 80 pp. 1–10
				

116
					Anon.Sir Gordon Holmes: A Neurologist of World ReputeTimesLondon30121965 p. 10
				

117 "The key claim of any transnational approach is its central concern with movements, flows, and circulation, not simply as a theme or motif, but as an analytic set of methods which defines the endeavor itself": HofmeyrIsabelAHR Conversation: On Transnational HistoryAm. Hist. Rev.2006111441–1464 p. 1444

